# Supplementary material for: Effects of bile salt-stimulated lipase on blood cells and associations with disease activity in human inflammatory joint disorders
Source: PLoS One. 2023 Aug 11;18(8):e0289980. doi: 10.1371/journal.pone.0289980 (PMC10420350; doi:10.1371/journal.pone.0289980)
Supplement: S1 Table — (DOCX) [file pone.0289980.s001.docx]

**S1 Table. Demographic data of the included patients with RA.**

|  | RA (n=15) |
| --- | --- |
| Age at diagnosis, mean (SD), years | 43.1 (14.0) |
| Females, n (%) | 9 (60) |
| Age at start of anti-TNF treatment, mean (SD), years | 54.5 (14.1) |
| Disease duration at anti-TNF start, mean (SD), years | 12.0 (9.9) |
| DAS28 at anti-TNF start, mean (SD) | 5.4 (1.2) |
| Mono/oligoarthritis at anti-TNF start, n (%) | - |
| Polyarthritis at anti-TNF start, n (%) | 15 (100) |
| Concomitant cDMARDs^1^ at TNF start, n (%) | 15 (100) |
| Methotrexate at anti-TNF start, n (%) | 13 (86.7) |

cDMARDs^1^ =conventional DMARDs (Methotrexate, azathioprine, leflunomide)
