# Supplementary material for: Effects of bile salt-stimulated lipase on blood cells and associations with disease activity in human inflammatory joint disorders
Source: PLoS One. 2023 Aug 11;18(8):e0289980. doi: 10.1371/journal.pone.0289980 (PMC10420350; doi:10.1371/journal.pone.0289980)
Supplement: S2 Table — (DOCX) [file pone.0289980.s002.docx]

**S2 Table. Demographic data of the included patients with PsA.**

|  | PsA (n= 43) |
| --- | --- |
| Age at diagnosis of PsA, mean (SD), years | 37.6 (15.8) |
| Females, n (%) | 19 (43) |
| Duration of Skin disease, mean (SD), years | 26.4 (14.8) |
| Duration of Joint disease, mean (SD), years | 16.8 (13.7) |
| Mono/oligoarthritis, n (%) | 22 (51) |
| Polyarthritis, n (%) | 21 (49) |
| Swollen and tender joint count, mean (SD) | 2.37 (2.57) |
| ESR, mm/h, mean (SD) | 14.1 (9.9) |
| hsCRP, mg/L, mean (SD) | 4.38 (4.60) |
| DMARD treatment, n (%) | 8 (18.6) |
