# Supplementary material for: Effects of bile salt-stimulated lipase on blood cells and associations with disease activity in human inflammatory joint disorders
Source: PLoS One. 2023 Aug 11;18(8):e0289980. doi: 10.1371/journal.pone.0289980 (PMC10420350; doi:10.1371/journal.pone.0289980)
Supplement: S3 Table — (DOCX) [file pone.0289980.s003.docx]

**S3 Table. Correlations between serum BSSL and disease associated parameters in PsA patients and healthy controls**

| **Parameter** | **Correlation coefficient (r_s_)** | **Sig. (2-tailed)** |
| --- | --- | --- |
| hs-CRP | 0.488 | <0.001 |
| S-calprotectin (S100A8/9) | 0.541 | <0.001 |
| Hemoglobin * | 0.315 | 0.040 |
| Leukocyte counts * | 0.242 | 0.117 |
| Thrombocyte counts * | 0.112 | 0.476 |
| ESR * | 0.176 | 0.265 |
| S-urate * | 0.079 | 0.619 |
| S-creatinine * | 0.025 | 0.874 |
| Disease duration * | 0.083 | 0.598 |
| Skin duration * | 0.001 | 0.993 |
| Il-12 | -0.088 | 0.464 |
| IL-15 | -0.012 | 0.920 |
| IL-16 | 0.036 | 0.768 |
| IL17A | -0.084 | 0.488 |
| IL-18 | -0.116 | 0.334 |
| IL-22 | 0.105 | 0.383 |
| IL-23 | 0.093 | 0.441 |
| IL-33 | 0.069 | 0.570 |
| CCL20 | 0.020 | 0.867 |
| CXCL10 | 0.103 | 0.392 |
| CXCL12 | -0.051 | 0.675 |

* Data based on blood samples or clinical examinations from PsA patients only.
