# Supplementary figures and images for: Effects of bile salt-stimulated lipase on blood cells and associations with disease activity in human inflammatory joint disorders
Source: PLoS One. 2023 Aug 11;18(8):e0289980. doi: 10.1371/journal.pone.0289980 (PMC10420350; doi:10.1371/journal.pone.0289980)

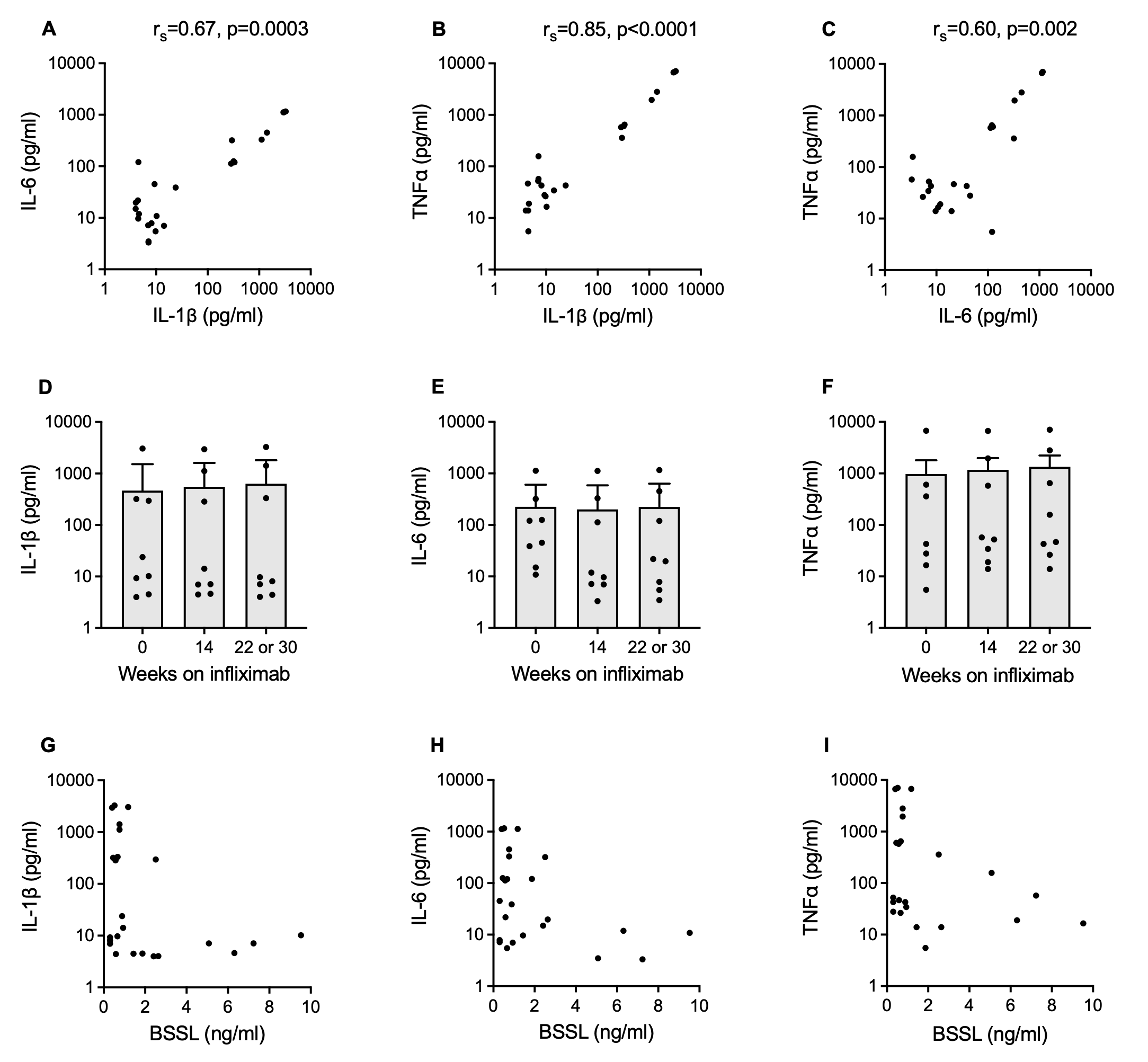

Supplement: S1 Fig — (A-C) Pariwise correlation between IL-1β, IL-6 and TNF⍺ plasma concentrations measured in 8 RA patients at three clinical visits each, i.e. in total 24 samples taken before the first infliximab infusion and then after 14 and 22 or 30 weeks of treatment. (D-F) IL-1β, IL-6 and TNF⍺ levels in plasma samples taken from 8 patients at each of three visits did not change significantly with duration of treament. Bars show mean ± SEM. (G-I) There was no correlation between BSSL plasma levels and any of the three cytokines analyzed. (TIFF) [file pone.0289980.s004.tiff]
