# Supplementary material for: Effects of bile salt-stimulated lipase on blood cells and associations with disease activity in human inflammatory joint disorders
Source: PLoS One. 2023 Aug 11;18(8):e0289980. doi: 10.1371/journal.pone.0289980 (PMC10420350; doi:10.1371/journal.pone.0289980)
Supplement: Attachment — Submitted filename: Response to Reviewers_230709.docx [file pone.0289980.s005.docx]

Dear Reviewer,

We appreciate the valuable comments and the possibility to resubmit a revised version of our manuscript entitled "Effects of bile salt-stimulated lipase on blood cells and associations with disease activity in human inflammatory joint disorders” (PONE-D-23-09819).

Please find our responses to each of the points raised by you below. With this revision, we hope that you will find the manuscript acceptable for publication.

Yours sincerely,

Susanne Lindquist

Reviewers’ Comments to Author:

**Reviewer #1:**

- Figure 8 is lacking in the manuscript.

**Response:** We apologize for not having noticed that Figure 8 was missing in the original submission. This figure is now included.

- In Figure 6A and Figure 7A and B, the regression curves need to be drawn, with confidence interval.

**Response:** The figures have been updated with nonparametric local regression curves (LOESS) and 95% confidence intervals.

- In Figure 6B/ and Figure 5 A to D, the group that are compared for statistical analyses need to be better evidenced.

**Response:** Connecting lines have been drawn to indicate which bars are compared.

- Figure 6C simply shows that DAS28 is reduced in treated patients...no need to put this part in the manuscript. eventually is the suppl materials.

**Response:** We agree, the figure 6C has been withdrawn.

- Figure 5D; the absence of effect of the WAY-121 inhibitor needs to be explained; While the KO as an effect in animal models, can a KI model, depleting enzymatic activity, confirm this chemical approach?

**Response:** The text in the discussion (first paragraph) has been slightly modified to try to clarify, and data showing the effect of WAY-121,898 on BSSLs enzymatic activity has been added in Fig 5 (E-F). The reviewer’s suggestion to develop a KI model, depleting enzymatic activity, is a great idea and certainly something that we will consider in our further research planning.

- Figure 3A. The absence of effect on granulocytes must be explained.

**Response:** Exogenous BSSL binds primarily to the surface of monocytes and only a weak binding was detected to granulocytes. Hence, although a minor (dose dependent) effect of mAb AS20 can be seen on the binding to granulocytes, we cannot expect an effect as clear as for monocytes.

- Figure 2A; confocal images must be shown to prove intracellular staining.

**Response:** Additional data with immunofluorescent staining’s and confocal images have been added to the manuscript (Fig 2). A new paragraph entitled “Immunofluorescence staining and confocal imaging” has been included in Material and Methods.

**Reviewer #2:**

The Manuscript by Lindquist et al., "Effects of bile salt-stimulated lipase on blood cells and associations with disease activity in human inflammatory joint disorders" tried to show the correlation between BSSL and arthritic diseases (RA and PsA). The study is preliminary to conclude the hypothesis and need further clarification and submission of missing data (figure 8).

- Did author look at the cytokines or chemokines after stimulations of CD14 cells with BSSL.

**Response:** This is certainly an interesting question that we currently address as part of a future manuscript. We ask for the reviewer´s understanding in this regard.

- Is there any reason to use different dose and values in fig 4 and 5 for BSSL? Keep the values consistent and also these 2 figures can be merged into one.

**Response:** In Fig 4 we use BSA as a control to show that the effect on cell migration is specific to BSSL, and not an unspecific effect of any unrelated protein at similar concentrations. In Fig 5 we investigate the capacity of different test items (antibodies and WAY-121,898) to interfere with BSSL induced cell migration. In these experiments the molar ratios between BSSL and test items are most relevant, and therefore we report concentrations in µM instead of µg/ml. A few changes have been made in the last paragraph on p 15 to clarify the relationship between protein concentration (µg/ml) and molarity, i.e., BSSL at 300 µg/ml = 4 µM.

- Is there any value range for BSSL in healthy vs RA or PsA?

**Response:** These data are shown in Fig 6 (RA) and Fig 8 (PsA and healthy). Fig 8 was unfortunately missing in the original submission but is now included.

- Fig 8 is missing from the manuscript.

**Response:** Again, we apologize for not having noticed that Figure 8 was missing in the original submission. This figure is now included.

- Reference 15 was used by large in whole manuscript.

**Response:** This point is well taken. However, to the best of our knowledge there are no other papers in the literature that describe the role of BSSL in animal models of inflammatory arthritis.
